# Labor Migration of Parents and Aggression Among Their Offspring in China

**DOI:** 10.1001/jamanetworkopen.2023.55315

**Published:** 2024-02-08

**Authors:** Ying Ma, Yanqi Li, Yi Zhang, Xinyi Xie, Xiaoyi Lin, Huihang Fu, Mengxin Huang, Weiju Zhou, John S. Ji, Ruoling Chen, Shun Liu, Yizhen Yu, Jie Tang

**Affiliations:** 1 Department of Child Healthcare, Guangzhou Women and Children’s Medical Center, Guangzhou Medical University, Guangzhou, PR China; 2 Department of Preventive Medicine, School of Public Health, Guangzhou Medical University, Xinzao, Panyu District, Guangzhou, PR China; 3 Vanke School of Public Health, Tsinghua University, Beijing, China; 4 Faculty of Education, Health & Wellbeing, University of Wolverhampton, Wolverhampton, United Kingdom; 5 Department of Adolescent & Maternal and Child Healthcare, School of Public Health, Guangxi Medical University, Qingxiu District, Nanning, PR China; 6 Department of Maternal and Child Healthcare, School of Public Health, Tongji Medical College, Huazhong University of Science and Technology, Hankou District, Wuhan, PR China

## Abstract

**Importance:**

In China, parental labor migration often leaves children behind and is potentially adversely associated with children’s health. However, the association between parental migration and aggression among their offspring remains largely underexplored.

**Objective:**

To investigate the associations of parental labor migration with total and subtypes of aggression among their offspring as well as potential sex differences therein.

**Design, Setting, and Participants:**

This cross-sectional study was conducted from February to October 2015 among students aged 11 to 20 years from 45 public middle and high schools across 5 provinces of China. Data analysis was performed from December 1, 2022, to August 1, 2023.

**Exposures:**

Parental migration, including migration status (yes or no), pattern (father, mother, or both), and the child’s age at the initial parent-child separation.

**Main Outcomes and Measures:**

Total and subtypes of aggression (including physical aggression [PA], verbal aggression [VA], indirect aggression [IA], anger, and hostility) were measured using the Chinese version of the Buss-Warren Aggression Questionnaire. Binomial logistic regression analyses were used separately to estimate adjusted odds ratios (aORs) and 95% CIs of parental migration by total and subtypes of aggression.

**Results:**

A total of 15 301 participants (7900 [51.6%] male and 7401 [48.4%] female) aged 11 to 20 years (mean [SD] age, 15.1 [1.8] years) were included. Of these participants, 5961 (39.0%) experienced parental migration; 2451 (16.0%) met the criteria for total aggression, 2407 (15.7%) for PA, 2283 (14.9%) for VA, 2899 (18.9%) for IA, 2307 (15.1%) for anger, and 2564 (16.8%) for hostility. Parental migration was associated with total aggression (aOR, 1.11; 95% CI, 1.01-1.22); no significant association between parental migration and subtypes of aggression was found except for PA (aOR, 1.14; 95% CI, 1.03-1.25). Compared with children whose parents did not migrate, the aORs for total aggression and PA for participants whose father migrated were 1.12 (95% CI, 1.01-1.28) and 1.15 (95% CI, 1.03-1.29), respectively; for participants with both parents having migrated, the aORs were 1.16 (95% CI, 1.02-1.31) and 1.12 (95% CI, 1.04-1.24), respectively. Compared with children whose parents did not migrate, children who experienced initial separation from 1 or both parents at adolescence had an aOR for total aggression of 1.20 (95% CI, 1.04-1.36), children who experienced initial separation from 1 or both parents at school age or adolescence had an aOR for PA of 1.15 (95% CI, 1.01-1.32) and 1.26 (95% CI, 1.04-1.51), respectively. No sex differences were found in these associations.

**Conclusions and Relevance:**

In this cross-sectional study of Chinese children and adolescents, we found that parental migration, mainly of the father or both parents or an initial separation at adolescence or school age from 1 or both parents, was associated with higher odds of total aggression and PA among offspring. These associations were similar between male and female participants.

## Key Points

**Question:**

Is parental labor migration in China associated with different types of aggression among children and adolescents who were left behind, and are there sex differences in this association?

**Findings:**

In this cross-sectional study of 15 301 youths, labor migration of the father or of both parents was associated with total and physical aggression, whereas differential associations by sex were not found. Additionally, higher odds of total and physical aggression were found among offspring who were initially separated from 1 or both parents at adolescence and school age.

**Meaning:**

These findings suggest that interventions targeting aggression should consider the potential associations with parental migration.

## Teaser

This cross-sectional study investigates the associations of parental labor migration in China with aggression among offspring overall and by sex.

## Introduction

Aggression refers to any activity with the immediate intent to cause harm, pain, or injury to another individual,^1^ including physical aggression (PA), verbal aggression (VA), indirect aggression (IA), anger, and hostility.^2^ Violent behavior is a form of PA with excessive harm to the target. Globally, aggressive and violent behavior is a serious public health concern and social problem among children and adolescents. It is estimated that 1 of 2 children aged 2 to 17 years experiences some form of aggressive and violent behavior, and emotional aggression affects 1 in 3 children.^3^ Data from the World Health Organization showed that aggressive and violent behaviors are responsible for 1.76 million deaths as well as millions of nonfatal injuries and disabilities every year.^4^ Over the course of a lifetime, children exposed to aggression are at increased risk of mental illness, high-risk behaviors, infectious diseases, chronic diseases, and further involvement in violence and crime.^3^ Low socioeconomic status is associated with an increased risk of aggression^5^; thus aggressive and violent behaviors may be more prevalent among rural Chinese children and adolescents given that studies have suggested that social economy in rural China is relatively low.^6^ A previous study^7^ suggested that approximately 24.3% of rural Chinese children and adolescents exhibited aggression. Thus, there is an urgent need to identify high-risk groups and specific risk factors for aggression among this population.

Labor migration refers to individuals who originate from low-waged areas and relocate for higher-waged employment opportunities. As a result of labor migration, the migrants’ household income often increases and their families’ circumstances improve. However, their children are often left behind in the care of other family members or caregivers owing to the transient nature of the work. Given these circumstances, numerous studies have examined the association between parental migration and health outcomes of children and adolescents.^8^ A comprehensive meta-analysis^8^ found that parental migration was associated with an increased risk of depression, anxiety, suicidal ideation, conduct disorders, substance use, wasting, and stunting among their offspring. Despite the findings, few studies have specifically focused on aggression.^9,10,11^ Findings that do exist are less convincing since these studies only compared risk of aggression between left-behind and non–left-behind children and adolescents by using univariate analysis methods.^9,11^ One study examining the independent association between parental labor migration and aggression among their offspring did not adjust for some important potential confounders,^10^ such as parenting style, social support, and emotional regulation factors.^12^ In addition, like other social-environmental factors (eg, childhood treatment),^13^ parental migration may have different impacts on total aggression and its subtypes. However, to our knowledge, no previous study has explored the possible associations of parental migration with the subtypes of aggression among their offspring. Therefore, the independent association of parental migration with total and subtypes of aggression remain uncertain.

One prior study suggested that a lack of parental support, the consequence for many left-behind children, had a greater association with hippocampal development at preschool age than at school age and early adolescence,^14^ implying that there may be sensitive periods for the effects of parent-child separation due to labor migration on health outcomes. However, to our knowledge, no study has explored whether the associations between parental migration and aggression may vary with the child’s age at the initial parent-child separation. It is of substantial public health importance to address these research gaps to inform strategies for aggression prevention and control, as well as to provide evidence for optimizing policies to protect labor migrants’ rights.

Our study aims to investigate the associations of parental labor migration, migration pattern, and the age of the child at the initial parent-child separation with total and subtypes of aggression among offspring. Since previous studies have shown sex differences in risk of total and subtypes of aggression^10,13^ and in the association of parental migration with health outcomes,^15^ we also aim to examine whether there are sex differences in the associations of parental migration with total and subtypes of aggression.

## Methods

### Study Participants

This study used the data from a nationwide cross-sectional study of 15 623 students from 45 middle and high schools across 5 provinces of China, which was conducted from February to October 2015. The design, procedure, and implementation of the nationwide cross-sectional study have been described previously (eMethods in Supplement 1).^16,17^ After excluding 30 participants who were orphans,^18^ 278 participants who did not respond to the items regarding parental migration, and 14 participants who did not complete an assessment of aggression with missing data for more than 10% of the items on aggression, we included 15 301 participants in the current analysis. This study followed the Strengthening the Reporting of Observational Studies in Epidemiology (STROBE) reporting guideline and received ethics clearance from Guangzhou Medical University and Huazhong University of Science and Technology. Before participation in the study, the students or their guardians (if students were younger than 14) provided written informed consent that was obtained in a manner consistent with the Declaration of Helsinki.^19^

### Instruments

Parental migration was categorized as yes based on the question, “Have/did your father or/and mother migrated to an urban area for employment and not living with you for at least half a year?”^20^ For those who answered yes to parental migration, parental migration pattern and the child’s age at the initial separation were investigated. The parental migration pattern was categorized into 3 groups (father, mother, or both parents) in response to the question, “Which of your parent(s) migrated to an urban area for employment and were not living with you for at least half a year?” The age of the child at the initial separation from 1 or both parents who migrated was categorized as preschool age (≤6 years), school age (6-10 years), or adolescence (>10 years) in response to the question “How old were you when 1 or both of your parents migrated to urban area for employment (the earliest time you know)?”

The Chinese version of the Buss-Warren Aggression Questionnaire (BWAQ) was used to assess total and subtypes of aggression.^21^ It consists of 34 items with 5-point Likert-type responses ranging from 1 (not at all like me) to 5 (completely like me). Raw scores for the total BWAQ and 5 subscales including PA, VA, IA, anger, and hostility were estimated first, then T scores placed on a scale with a mean of 50 and an SD of 10 were calculated.^21^ Higher raw scores and T scores indicate higher levels of total and subtypes of aggression. Participants were grouped into 7 groups according to T scores based on the cutoff point proposed in a previous study conducted among Chinese adolescents^22^: very low (T score ≤29), low (T score 30-39), low average (T score 40-44), average (T score 45-55), high average (T score 56-59), high (T score 60-69), and very high (T score ≥70). In the current study, we defined high and very high groups as exhibiting corresponding aggression, while other groups were defined as no corresponding aggression.^21^ The BWAQ has good internal consistency among Chinese children and adolescents.^13^ The Cronbach α for total BWAQ and 5 subscales in the study was 0.90, and 0.85, 0.61, 0.72, 0.74 and 0.73, respectively.

We constructed a direct acyclic graph (DAG; eFigure in Supplement 1) to select potential confounders when examining the association between parental migration and aggression among their offspring. Specifically, we selected study province (Heilongjiang, Hubei, Anhui, Guangdong, and Yunnan), age (continuous), sex (male or female), child’s ethnicity (Han or others), single-parent family (yes or no), single-child family (yes or no), family income (<$150/mo, $150-$850/mo, or >$850/mo),^16^ educational level of the main caregiver (middle school or below, high school or technical school, college or above), parenting style (strict, pampered, neglect or frequently changing, or open-minded),^23^ social support (continuous),^24^ loneliness (continuous),^25^ psychological resilience (continuous),^26^ and emotional management ability (continuous)^27^ as the potential confounders. Information on measurements of these confounders can be found in eMethods in Supplement 1.

### Statistical Analysis

The data analysis was performed from December 1, 2022, to August 1, 2023. Binomial logistic regression models were used to estimate the adjusted odds ratios (aORs) and 95% CIs of parental migration for participants with total or subtypes of aggression, and different confounders were adjusted in 3 models to examine the robustness of our findings. In the secondary analyses, the same regression models were used to separately examine the associations of parental migration pattern (ie, which parent migrated) and child age at initial child-parent separation with total or subtypes of aggression.

We conducted sensitivity analyses by defining total and subtypes of aggression with a more rigorous criterion (ie, categorized the very high [T score ≥70T] group as exhibiting corresponding aggression). We further conducted subgroup analyses to examine whether the associations of parental migration with total or subtypes of aggression differed by sex. Differences were assessed by calculating a ratio of the ORs.^28,29^

The statistical significance level for primary analyses was *P* < .05. To reduce the potential for type I errors due to multiple comparisons in secondary and subgroup analyses, we adjusted the statistical significance level using the Bonferroni method.^30^ All tests were 2-sided unless otherwise specified. All analyses were conducted using SPSS Statistics, version 26.0 (IBM Corp). Additional information on the statistical analysis can be found in eMethods in Supplement 1.

## Results

The analytic sample of 15 301 participants (7900 [51.6%] male and 7401 [48.4%] female) were approximately equally distributed across the 5 study provinces. The age of the participants ranged from 11 to 20 years (mean [SD] age, 15.1 [1.8] years). Most were Han ethnicity (13 849 [90.5%]), 5286 (34.5%) were a single child, and 752 (4.9%) were from single-parent families. In total, 5961 participants (39.0%) experienced parental migration; 3573 (23.4%) experienced migration of the father, 480 (3.1%) experienced migration of the mother, and 1908 (12.5%) experienced migration of both parents. The percentages of participants who were initially separated their parents were 17.4% (2663 of 15 301) at preschool age (≤6 years), 15.2% (2330 of 15 301) at school age (6-10 years), and 6.3% (968 of 15 301) at adolescents (>10 years). Additional characteristics are presented in Table 1.

**Table 1.** Characteristics of Participants by the Presence or Absence of Parental Migration

Characteristic^a^	Participants, No. (%)								
	Total (N = 15301)	Parental migration		Total aggression (n = 2451)	Subtypes of aggression				
		No (n = 9340)	Yes (n = 5961)		PA (n = 2407)	VA (n = 2283)	IA (n = 2899)	Anger (n = 2307)	Hostility (n = 2564)
Age, mean (SD), y	15.1 (1.8)	15.1 (1.8)	15.2 (1.9)	15.2 (1.8)	15.1 (1.8)	15.2 (1.8)	15.3 (1.8)	15.2 (1.8)	15.2 (1.8)
Sex									
Male	7900 (51.6)	4791 (51.3)	3109 (52.2)	1359 (55.4)	1628 (67.6)	1312 (57.5)	1491 (51.4)	1043 (45.2)	1326 (51.7)
Female	7401 (48.4)	4549 (48.7)	2852 (47.8)	1092 (44.6)	779 (32.4)	971 (42.5)	1408 (48.6)	1264 (54.8)	1238 (48.3)
Province									
Anhui	3302 (21.6)	1459 (15.6)	1843 (30.9)	559 (22.8)	551 (22.9)	542 (23.7)	634 (21.9)	513 (22.2)	567 (22.1)
Guangdong	2979 (19.5)	1873 (20.1)	1106 (18.6)	402 (16.4)	286 (11.9)	415 (18.2)	540 (18.6)	404 (17.5)	562 (21.9)
Heilongjiang	2750 (18.0)	2045 (21.9)	705 (11.8)	422 (17.2)	453 (18.8)	444 (19.4)	472 (16.3)	413 (17.9)	402 (15.7)
Hubei	2939 (19.2)	1290 (13.8)	1649 (27.7)	437 (17.8)	461 (19.2)	380 (16.6)	604 (20.8)	367 (15.9)	452 (17.6)
Yunnan	3331 (21.8)	2673 (28.6)	658 (11.0)	631 (25.7)	656 (27.3)	502 (22.0)	649 (22.4)	610 (26.4)	581 (22.7)
Educational level									
Junior high school	8153 (53.3)	5210 (55.8)	2943 (49.4)	1225 (50.0)	1284 (53.3)	1204 (52.7)	1379 (47.6)	1201 (52.1)	1302 (50.8)
Senior high school	7148 (46.7)	4130 (44.2)	3018 (50.6)	1226 (50.0)	1123 (46.7)	1079 (47.3)	1520 (52.4)	1106 (17.9)	1262 (49.2)
Ethnicity									
Han	13 849 (90.5)	8162 (87.4)	5687 (95.4)	2151 (87.8)	2095 (87.0)	2056 (90.1)	2607 (89.9)	2012 (87.2)	2286 (89.2)
Other^b^	1452 (9.5)	1178 (12.6)	274 (4.6)	300 (12.2)	312 (13.0)	227 (9.9)	292 (10.1)	295 (12.8)	278 (10.8)
Educational level of main caregiver^c^									
Junior middle school or below	10 745 (70.2)	6038 (64.7)	4707 (79.0)	1679 (68.5)	1663 (69.1)	1612 (70.6)	1991 (68.7)	1584 (68.7)	1790 (69.8)
Senior middle school or technical school	3277 (21.4)	2258 (24.2)	1019 (17.1)	564 (23.0)	547 (22.7)	494 (21.6)	658 (22.7)	527 (22.8)	563 (22.0)
College or above	1052 (6.9)	882 (9.4)	170 (2.8)	174 (7.1)	164 (6.8)	156 (6.9)	203 (7.0)	163 (7.1)	174 (6.8)
Missing data	227 (1.5)	162 (1.7)	65 (1.1)	34 (1.4)	33 (1.4)	21 (0.9)	47 (1.6)	33 (1.4)	37 (1.4)
Single-child family	5286 (34.5)	3703 (39.6)	1583 (26.6)	853 (34.8)	898 (37.3)	795 (34.8)	1051 (36.3)	775 (33.6)	843 (32.9)
Single-parent family	752 (4.9)	407 (4.4)	345 (5.8)	127 (5.2)	125 (5.2)	120 (5.3)	142 (4.9)	122 (5.3)	139 (5.4)
Family income, $/mo									
<150	2643 (17.3)	1614 (17.3)	1029 (17.3)	428 (17.5)	422 (17.3)	355 (15.5)	471 (16.2)	383 (16.6)	431 (16.8)
150-850	10 625 (69.4)	6464 (69.2)	4161 (69.8)	1643 (67.0)	1576 (65.5)	1603 (70.2)	2010 (69.3)	1562 (67.7)	1791 (69.9)
>850	2033 (13.3)	1262 (13.5)	771 (12.9)	380 (15.5)	409 (17.0)	325 (14.2)	418 (14.4)	362 (15.7)	342 (13.3)
Parenting style^c^									
Strict	4640 (30.3)	2871 (30.7)	1769 (29.7)	720 (29.4)	742 (30.8)	721 (31.6)	734 (25.3)	713 (30.9)	757 (29.5)
Pamper	547 (3.6)	294 (3.2)	253 (4.2)	139 (5.7)	143 (5.9)	107 (4.7)	154 (5.3)	129 (5.6)	112 (4.4)
Neglect	1645 (10.8)	936 (10.0)	709 (11.9)	449 (18.3)	402 (16.8)	318 (13.9)	441 (15.2)	367 (15.9)	460 (17.9)
Open minded	7854 (51.3)	4859 (52.0)	2995 (50.3)	1049 (42.8)	1019 (42.3)	1052 (46.1)	1435 (49.5)	1013 (43.9)	1141 (44.5)
Missing data	615 (4.0)	380 (4.1)	235 (3.9)	94 (3.8)	101 (4.2)	85 (3.7)	135 (4.7)	85 (3.7)	94 (3.7)
Loneliness score, mean (SD)	49.9 (10.2)	49.3 (10.1)	50.8 (10.4)	56.9 (11.3)	54.3 (11.2)	53.7 (10.7)	54.1 (10.9)	55.0 (11.2)	57.5 (11.3)
Psychological resilience score, mean (SD)	92.1 (13.1)	92.7 (13.1)	91.1 (12.9)	86.2 (12.3)	87.6 (12.3)	92.0 (13.1)	88.4 (12.5)	86.7 (12.5)	85.8 (12.3)
Emotional management ability score, mean (SD)	11.5 (2.8)	11.6 (2.8)	11.4 (2.9)	9.6 (3.0)	10.3 (3.1)	107 (3.1)	10.4 (3.0)	9.6 (3.1)	9.8 (3.0)
Social support score, mean (SD)	63.1 (14.3)	63.8 (14.2)	62.1 (14.4)	58.0 (14.7)	58.9 (14.5)	62.0 (14.6)	59.8 (14.6)	59.7 (14.9)	56.8 (14.7)

Abbreviations: IA, indirect aggression; PA, physical aggression; VA, verbal aggression.

^a^ The distributions of parental migration with these characteristics were all statistically significant (*P* < .05), except for sex and family income.

^b^ Includes all ethnicities except Han.

^c^ There were missing data for this characteristic.

Of 15 301 participants, 2451 (16.0%) met the criteria for total aggression. Additionally, 2407 (15.7%) met the criteria for PA, 2283 (14.9%) for VA, 2899 (18.9%) for IA, 2307 (15.1%) for anger, and 2564 (16.8%) for hostility (Table 1).

Participants who experienced parental migration had an increased risk of total aggression, PA, and hostility (Table 2). Significant associations were found between parental migration status (ie, whether a parent migrated) and total aggression or PA in all models. In the fully adjusted model (model 3), the aOR for parental migration was 1.11 (95% CI, 1.01-1.22) for total aggression and 1.14 (95% CI, 1.03-1.25) for PA. Associations between parental migration and hostility were found in unadjusted model and model 1. However, the association disappeared with adjustment for confounders of family-level characteristics and psychological variables. No significant associations of parental migration with VA, IA, or anger were found in any model (Table 2). In subgroup analysis, no significant sex differences were observed in the associations of parental migration with total or subtypes of aggression (eTable 1 in Supplement 1).

**Table 2.** Odds of Total and Subtype of Aggression by Parental Migration Status

Variable	Participants, No. (%)	OR (95% CI)			
		Unadjusted	Model 1^a^	Model 2^b^	Model 3^c^
**Total aggression**					
No migration	1439 (15.4)	1 [Reference]	1 [Reference]	1 [Reference]	1 [Reference]
Migration	1012 (17.0)	1.12 (1.03-1.23)	1.18 (1.08-1.30)	1.13 (1.03-1.24)	1.11 (1.01-1.22)
**PA**					
No migration	1413 (15.1)	1 [Reference]	1 [Reference]	1 [Reference]	1 [Reference]
Migration	994 (16.7)	1.12 (1.03-1.23)	1.21 (1.10-1.33)	1.18 (1.07-1.30)	1.14 (1.03-1.25)
**VA**					
No migration	1395 (14.9)	1 [Reference]	1 [Reference]	1 [Reference]	1 [Reference]
Migration	888 (14.9)	1.00 (0.91-1.09)	1.02 (0.92-1.12)	0.97 (0.88-1.06)	0.94 (0.85-1.03)
**IA**					
No migration	1757 (18.8)	1 [Reference]	1 [Reference]	1 [Reference]	1 [Reference]
Migration	1142 (19.2)	1.02 (0.94-1.11)	1.03 (0.94-1.12)	1.01 (0.93-1.11)	0.97 (0.89-1.06)
**Anger**					
No migration	1413 (15.1)	1 [Reference]	1 [Reference]	1 [Reference]	1 [Reference]
Migration	894 (15.0)	1.02 (0.97-1.11)	1.06 (0.96-1.17)	1.00 (0.91-1.10)	0.93 (0.84-1.03)
**Hostility**					
No migration	1515 (16.2)	1 [Reference]	1 [Reference]	1 [Reference]	1 [Reference]
Migration	1049 (17.6)	1.10 (1.01-1.20)	1.12 (1.03-1.23)	1.05 (0.96-1.15)	0.98 (0.88-1.08)

Abbreviations: IA, indirect aggression; OR, odds ratio; PA, physical aggression; VA, verbal aggression.

^a^ Adjusted for province, age, sex, and ethnicity.

^b^ Additionally adjusted for single-child family, single-parent family, educational level of main caregiver, family income, parenting style, and social support.

^c^ Additionally adjusted for offspring loneliness, psychological resilience, and emotional management ability scores.

Compared with participants with parents who did not migrate, participants with migration of the father or both parents but not with migration of the mother had significantly increased both unadjusted ORs and aORs for total aggression and PA (Table 3). In the fully adjusted model for total aggression (model 3), the aOR was 1.12 (95% CI, 1.01-1.28) for migration of the father, 1.16 (95% CI, 1.02-1.31) for migration of both parents, and 1.04 (95% CI, 0.79-1.37) for migration of the mother. For PA, the aOR was 1.15 (95% CI, 1.03-1.29) for migration of the father, 1.12 (95% CI, 1.04-1.24) for migration of both parents, and 1.11 (95% CI, 0.85-1.45) for migration of the mother. There was no significant association between which parent migrated and VA, IA, anger, or hostility. Additionally, no sex differences were observed in the associations of which parent migrated with total or subtypes of aggression (eTable 2 in Supplement 1).

**Table 3.** Odds of Total and Subtype of Aggression by Parental Migration Type

Variable	Participants, No. (%)	OR (95% CI)			
		Unadjusted	Model 1^a^	Model 2^b^	Model 3^c^
**Total aggression**					
None	1439 (15.4)	1 [Reference]	1 [Reference]	1 [Reference]	1 [Reference]
Father	598 (16.7)	1.11 (1.01-1.23)	1.15 (1.04-1.28)	1.14 (1.02-1.27)	1.12 (1.01-1.28)
Mother	75 (15.6)	1.02 (0.79-1.31)	1.04 (0.81-1.32)	1.04 (0.80-1.34)	1.04 (0.79-1.37)
Both parents	339 (17.8)	1.19 (1.04-1.35)	1.23 (1.08-1.40)	1.19 (1.04-1.34)	1.16 (1.02-1.31)
**PA**					
None	1413 (15.1)	1 [Reference]	1 [Reference]	1 [Reference]	1 [Reference]
Father	600 (16.8)	1.13 (1.02-1.26)	1.22 (1.10-1.36)	1.21 (1.09-1.35)	1.15 (1.03-1.29)
Mother	75 (15.6)	1.04 (0.81-1.34)	1.10 (0.85-1.42)	1.11 (0.86-1.44)	1.11 (0.85-1.45)
Both parents	319 (16.7)	1.13 (1.04-1.23)	1.21 (1.06-1.39)	1.14 (1.10-1.31)	1.12 (1.04-1.24)
**VA**					
None	1395 (14.9)	1 [Reference]	1 [Reference]	1 [Reference]	1 [Reference]
Father	539 (15.1)	1.01 (0.91-1.13)	1.00 (0.90-1.12)	0.99 (0.88-1.10)	0.96 (0.85-1.07)
Mother	68 (14.2)	0.94 (0.72-1.22)	0.93 (0.71-1.21)	0.92 (0.70-1.20)	0.92 (0.70-1.20)
Both parents	281 (14.7)	0.98 (0.86-1.13)	0.97 (0.84-1.12)	0.95 (0.83-1.09)	0.91 (0.79-1.05)
**IA**					
None	1757 (18.8)	1 [Reference]	1 [Reference]	1 [Reference]	1 [Reference]
Father	676 (18.9)	1.01 (0.91-1.11)	1.02 (0.92-1.13)	1.03 (0.93-1.14)	0.97 (0.87-1.08)
Mother	77 (20.4)	0.83 (0.64-1.06)	0.83 (0.64-1.06)	0.82 (0.64-1.06)	0.81 (0.63-1.05)
Both parents	389 (18.9)	1.11 (0.98-1.25)	1.09 (0.97-1.24)	1.04 (0.92-1.18)	1.01 (0.88-1.15)
**Anger**					
None	1413 (15.1)	1 [Reference]	1 [Reference]	1 [Reference]	1 [Reference]
Father	530 (14.8)	0.98 (0.88-1.09)	1.01 (0.91-1.13)	1.00 (0.89-1.12)	0.92 (0.82-1.04)
Mother	66 (13.8)	0.90 (0.69-1.17)	0.91 (0.70-1.19)	0.91 (0.69-1.19)	0.90 (0.68-1.20)
Both parents	298 (15.6)	1.04 (0.91-1.19)	1.08 (0.94-1.24)	1.01 (0.88-1.17)	0.96 (0.83-1.12)
**Hostility**					
None	1513 (16.2)	1 [Reference]	1 [Reference]	1 [Reference]	1 [Reference]
Father	624 (17.5)	1.10 (0.99-1.21)	1.10 (0.99-1.22)	1.06 (0.96-1.18)	0.97 (0.87-1.09)
Mother	78 (16.3)	1.01 (0.78-1.29)	1.00 (0.78-1.28)	0.97 (0.75-1.26)	0.97 (0.74-1.27)
Both parents	347 (18.2)	1.15 (1.01-1.31)	1.16 (1.02-1.32)	1.05 (0.92-1.20)	0.98 (0.85-1.14)

Abbreviations: IA, indirect aggression; OR, odds ratio; PA, physical aggression; VA, verbal aggression.

^a^ Adjusted for province, age, sex, and ethnicity.

^b^ Additionally adjusted for single-child family, single-parent family, educational level of main caregiver, family income, parenting style, and social support.

^c^ Additionally adjusted for offspring loneliness, psychological resilience, and emotional management ability scores.

Participants who experienced initial separation from 1 or both parents before the age 6 years, between the ages of 6 and 10 years, or after the age of 10 years reported a higher likelihood of being at risk for total aggression than participants whose parents did not migrate (Table 4). However, in the fully adjusted model (model 3), significant associations were found only among participants who experienced initial separation from migrating parents after the age of 10 years (aOR, 1.20; 95% CI, 1.04-1.36). The unadjusted ORs and aORs in model 1 and model 2 for PA were also significantly higher among participants who experienced initial separation before the age of 6, between the age of 6 and 10, or over the age of 10 from 1 or both parents who migrated. However, in the fully adjusted model (model 3), significant associations were found only among participants who experienced initial separation between ages 6 and 10 years (aOR, 1.15; 95% CI, 1.01-1.32) or after age 10 years (aOR, 1.26; 95% CI, 1.04-1.51). Subgroup analysis also showed that there were also no significant sex differences in the associations of initial separation from 1 or both migrating parents with total and subtypes of aggression (eTable 3 in Supplement 1).

**Table 4.** Odds of Total and Subtype of Aggression by Age of Offspring When Parent Initially Migrated

Variable	Participants, No. (%)	OR (95% CI)			
		Unadjusted	Model 1^a^	Model 2^b^	Model 3^c^
**Total aggression**					
No migration	1356 (15.5)	1 [Reference]	1 [Reference]	1 [Reference]	1 [Reference]
Preschool age (≤6 y)	495 (17.2)	1.16 (1.04-1.30)	1.22 (1.09-1.37)	1.15 (1.02-1.30)	1.01 (0.89-1.16)
School age (6-10 y)	400 (15.9)	1.06 (0.94-1.20)	1.11 (0.98-1.25)	1.08 (0.95-1.22)	1.06 (0.92-1.21)
Adolescence (>10 y)	200 (17.3)	1.17 (0.98-1.40)	1.18 (1.01-1.39)	1.18 (1.02-1.38)	1.20 (1.04-1.36)
**PA**					
No migration	1306 (14.9)	1 [Reference]	1 [Reference]	1 [Reference]	1 [Reference]
Preschool age (≤6 y)	471 (16.4)	1.10 (0.98-1.24)	1.21 (1.08-1.37)	1.16 (1.03-1.32)	1.08 (0.95-1.23)
School age (6-10 y)	420 (16.7)	1.12 (1.03-1.30)	1.19 (1.05-1.35)	1.17 (1.03-1.33)	1.15 (1.01-1.32)
Adolescence (>10 y)	210 (18.2)	1.18 (1.02-1.43)	1.24 (1.04-1.49)	1.25 (1.04-1.50)	1.26 (1.04-1.51)
**VA**					
No migration	1305 (14.9)	1 [Reference]	1 [Reference]	1 [Reference]	1 [Reference]
Preschool age (≤6 y)	428 (14.9)	0.99 (0.87-1.11)	0.98 (0.87-1.11)	0.96 (0.84-1.08)	0.90 (0.79-1.02)
School age (6-10 y)	373 (14.8)	1.00 (0.88-1.14)	0.99 (0.87-1.12)	0.97 (0.85-1.11)	0.96 (0.84-1.10)
Adolescence (>10 y)	177 (15.4)	1.02 (0.85-1.23)	1.01 (0.83-1.21)	1.00 (0.83-1.20)	0.99 (0.82-1.19)
**IA**					
No migration	1658 (18.9)	1 [Reference]	1 [Reference]	1 [Reference]	1 [Reference]
Preschool age (≤6 y)	571 (19.9)	1.07 (0.96-1.20)	1.08 (0.97-1.21)	1.05 (0.94-1.18)	0.98 (0.87-1.09)
School age (6-10 y)	448 (17.8)	0.96 (0.86-1.08)	0.97 (0.87-1.10)	0.97 (0.86-1.09)	0.94 (083-1.07)
Adolescence (>10 y)	222 (19.3)	1.04 (0.88-1.23)	1.01 (0.86-1.20)	1.03 (0.87-1.22)	1.02 (0.86-1.21)
**Anger**					
No migration	1328 (15.2)	1 [Reference]	1 [Reference]	1 [Reference]	1 [Reference]
Preschool age (≤6 y)	436 (15.2)	1.02 (0.90-1.15)	1.05 (0.93-1.19)	1.00 (0.89-1.14)	0.89 (0.78-1.02)
School age (6-10 y)	366 (14.5)	0.97 (0.85-1.10)	1.01 (0.89-1.15)	0.99 (0.87-1.13)	0.96 (0.84-1.10)
Adolescence (>10 y)	177 (15.4)	1.02 (0.87-1.17)	1.01 (0.89-1.13)	1.00 (0.82-1.19)	0.98 (0.80-1.19)
**Hostility**					
No migration	1409 (16.1)	1 [Reference]	1 [Reference]	1 [Reference]	1 [Reference]
Preschool age (≤6 y)	536 (18.6)	1.18 (1.05-1.32)	1.19 (1.06-1.33)	1.09 (0.97-1.23)	0.95 (0.84-1.08)
School age (6-10 y)	413 (16.4)	1.01 (0.90-1.15)	1.02 (0.90-1.16)	0.97 (0.86-1.10)	0.94 (0.82-1.08)
Adolescence (>10 y)	206 (17.9)	1.14 (0.96-1.35)	1.13 (0.95-1.34)	1.14 (0.95-1.36)	1.14 (0.94-1.37)

Abbreviations: IA, indirect aggression; OR, odds ratio; PA, physical aggression; VA, verbal aggression.

^a^ Adjusted for province, age, sex, and ethnicity.

^b^ Additionally adjusted for single-child family, single-parent family, educational level of main caregiver, family income, parenting style, and social support.

^c^ Additionally adjusted for offspring loneliness, psychological resilience, and emotional management ability scores.

In sensitivity analysis, we used a rigorous criterion to define aggression (ie, categorized the very high group as exhibiting aggression). Similar significant associations between parental labor migration (including migration status, migration pattern, and initial age when parents migrated) and aggression among their offspring were found (eTables 4-6 in Supplement 1).

## Discussion

This study found that parental migration was positively associated with total aggression and PA but not VA, IA, anger, or hostility. The data also suggested that migration of the father or both parents, rather than migration of the mother, was associated with total aggression and PA. Offspring initially separated at adolescence (>10 years) from 1 or both migrating parents were more likely to report total aggression or PA than offspring who initially separated at school age (6-10 years) and preschool age (≤6 years). No significant sex differences were found in the associations of parental migration status, migration pattern, and child age at initial separation with total and subtypes of aggression. These findings are important to possibly inform the development of preventive interventions for aggression among children and adolescents and the policies to support migrating families.

Although several studies examined risk factors associated with aggression among left-behind children and adolescents,^31,32,33^ only 3 studies have examined the association between parental migration and aggression, with less convincing findings due to the use of an unsuitable analysis strategy^9,11^ or the failure to adjust for some important potential confounders.^10^ In the present study, we used a rational data analysis strategy and adjusted for as many as 13 potential confounders, providing robust evidence that parental migration is independently associated with an increased risk of total aggression among Chinese children and adolescents and that those offspring who were left behind are at high risk of total aggression.

Our findings suggest that parental migration is independently associated with an increased risk of PA but not with other subtypes of aggression. These results were inconsistent with previous studies,^9,10^ which suggested significant differences in the risks of all subtypes of aggression between left-behind and non–left-behind children. The discrepancies in the findings between studies for the associations of parental migration with subtypes of aggression may be related to the study location apart from study design, study sample, and adjustments. In China, the health and well-being of left-behind children has been a priority concern over the past 2 decades.^34^ The Chinese government has been calling on local authorities to take responsibility for taking care of these children.^35^ However, health care policies and actions for left-behind children vary across provinces, making it difficult to ascertain their associations with the health and well-being of these children.^36^ The risk of aggression among left-behind children may be mitigated in places with well-formulated health care policies and actions (eTable 7 in Supplement 1). In addition, previous studies indicated that subtypes of aggression have different forms and functions (ie, social vs physical aggression; reactive vs proactive aggression) and thus may have different correlates and etiologies.^37,38^ Given China’s ongoing actions for left-behind children and the differing correlates and etiologies for subtypes of aggression, it is perhaps not surprising to see the nonsignificant results of parental migration with VA, IA, anger, and hostility.

To our knowledge, no previous study has investigated sex differences in the association between parental migration and aggression, but several studies have examined sex differences in the association between parent-child relationship, the proxy of parental migration, and aggression.^39,40^ For example, a study of the Global School–based Student Health Survey found that male children with a poor bond with their parents had 2 times higher prevalence of PA compared with female children with the strong parent-child bonding,^39^ which was inconsistent with our findings. Traditional Chinese culture tends to favor sons over daughters, particularly in rural China. Under such a background, males are often perceived as dominant, competitive, and impulsive in adolescence. In the absence of parental supervision, male children may be more susceptible to negative influences from school or social environments, leading them to prefer PA toward peers or other people. On the contrary, female children are socialized to be compliant, subordinate, and overcontrolling, which renders them more vulnerable to emotional difficulties and more prone to exhibiting reactive aggression through regulatory emotional self-efficacy and emotion regulation.^41,42^ Given these complexities and potential cultural differences, further investigations are warranted to verify whether there are sex differences in the associations of parental migration with total and subtypes of aggression.

The associations of parental migration with total aggression and PA may be explained as follows. First, the caretakers of left-behind children often have different family roles, parenting styles, educational levels, and lifestyles from the children’s own parents, which may be risk factors associated with an unfavorable environment for psychological development related to aggression, such as attachment insecurity, poor emotional regulation, weak self-control, and reduced sociability.^40,43^ Second, compared with migration of the mother, migration of the father appeared to have stronger association with total aggression and PA, possibly due to the different caregiving roles of fathers and mothers.^44^ Previous studies have shown that a mother’s care can contribute to the development of a careful and sensitive personality in children, while a father’s care was crucial in fostering an independent and strong personality, which was associated with aggression development.^45^ Moreover, migrating fathers were also associated with providing less warmth and emotional support than migrating mothers.^46^ Third, an initial separation at adolescence from 1 or both parents was more strongly associated with total aggression and/or PA than separation at school age and/or preschool age. This finding may be because adolescence is a critical period of neural development and the shaping of behavioral trajectories, making it a vulnerable period during which developmental processes may be easily disrupted.^2^ Further studies are warranted to delineate specific developmental mechanisms and their associations with parental migration on aggression.

### Limitations

This study has limitations. First, this study used a cross-sectional design, implying that the direction of the association between parental migration and aggression among the offspring cannot be conclusively inferred. Second, parental migration may be misclassified, and the prevalence of total and subtypes of aggression may be underestimated or overestimated owing to recall bias. Nonetheless, the proportion of left-behind children in our study was comparable with that in the monitoring and investigation report on migrant work in China.^47^ Additionally, numerous studies have indicated that school-based self-reported data collection regarding behavior and psychology and correlates is likely to be reliable, and such data are valuable when prospective data are not available.^48^ Third, although study investigators were trained to use a standardized procedure to collect data, participants may not have the same capacity to understand all the questionnaire items because the age span of the participants is large. Fourth, although we conducted secondary analysis to examine the association between the timing of the parent-child separation and aggression, we did not consider the effects of duration of parent-child separation since we did not collect this metric in the survey.^9^ Fifth, although we adjusted for many potential confounders, we cannot rule out the possibility of residual confounders by unknown and other factors that were not assessed in our survey. Sixth, although we adjusted for social support and parenting style, we did not examine the mediating and moderating mechanisms underlying the association between parental migration and aggression, which will be the direction of our future studies.

## Conclusions

Findings of this cross-sectional study indicated an independent association between parental migration and total aggression and PA among their offspring. This emphasizes the necessity to monitor aggression and its associated risk factors among left-behind children and adolescents and to implement early interventions that offer tailored support strategies. Children who experienced the migration of the father or both parents and those who underwent separation at adolescence or school age from 1 or both migrant parents may be vulnerable. These insights can inform teachers, health educators, and other stakeholders about the potential health needs of left-behind children, thereby contributing to the formulation of specific policy initiatives aimed at enhancing this population’s behavioral health. Future research is necessary to explore potential sex differences in the association between parental migration and various forms of aggression. This could further aid in the development of a multidimensional intervention framework to address aggression among children and adolescents left behind due to parental labor migration.

## Notes

Supplement 1.
eMethods.
**eFigure.** Directed Acyclic Graph for the Association Between Parental Migration and Aggression Among Their Offspring **eTable 1.** Total and Subtypes of Aggression by Migration Status in Male vs Female Participants **eTable 2.** Total and Subtypes of Aggression by Parental Migration Type in Male vs Female Participants **eTable 3.** Total and Subtypes of Aggression by Parental Migration Stage in Male vs Female Participants **eTable 4.** Odds of Severe Aggression by Parental Migration Status **eTable 5.** Odds of Severe Aggression by Parental Migration Type **eTable 6.** Odds of Severe Aggression by Age of Offspring When Parent Initially Migrated **eTable 7.** Association of Parental Migration Status With Total and Subtypes of Aggression by Study Location

Supplement 2.
Data Sharing Statement
