# Supplementary material for: Labor Migration of Parents and Aggression Among Their Offspring in China
Source: JAMA Netw Open. 2024 Feb 8;7(2):e2355315. doi: 10.1001/jamanetworkopen.2023.55315 (PMC10853836; doi:10.1001/jamanetworkopen.2023.55315)
Supplement: Supplement 1. — eMethods. eFigure. Directed Acyclic Graph for the Association Between Parental Migration and Aggression Among Their Offspring eTable 1. Total and Subtypes of Aggression by Migration Status in Male vs Female Participants eTable 2. Total and Subtypes of Aggression by Parental Migration Type in Male vs Female Participants eTable 3. Total and Subtypes of Aggression by Parental Migration Stage in Male vs Female Participants eTable 4. Odds of Severe Aggression by Parental Migration Status eTable 5. Odds of Severe Aggression by Parental Migration Type eTable 6. Odds of Severe Aggression by Age of Offspring When Parent Initially Migrated eTable 7. Association of Parental Migration Status With Total and Subtypes of Aggression by Study Location [file jamanetwopen-e2355315-s001.pdf]

Supplemental Online Content

Ma Y, Li Y, Zhang Y, et al. Labor migration of parents and aggression among their offspring in China. *JAMA Netw Open.* 2024;7(2):e2355315.
doi:10.1001/jamanetworkopen.2023.55315

eMethods.

eFigure. Directed Acyclic Graph for the Association Between Parental Migration and Aggression Among Their Offspring

eTable 1. Total and Subtypes of Aggression by Migration Status in Male vs Female Participants

eTable 2. Total and Subtypes of Aggression by Parental Migration Type in Male vs Female Participants

eTable 3. Total and Subtypes of Aggression by Parental Migration Stage in Male vs Female Participants

eTable 4. Odds of Severe Aggression by Parental Migration Status

eTable 5. Odds of Severe Aggression by Parental Migration Type

eTable 6. Odds of Severe Aggression by Age of Offspring When Parent Initially Migrated

eTable 7. Association of Parental Migration Status With Total and Subtypes of Aggression by Study Location

This supplemental material has been provided by the authors to give readers additional information about their work.

© 2024 Ma Y et al. *JAMA Network Open.*

eMethods.

Design, procedure, and implementation of the nationwide cross-sectional study

Study aims: To investigate the epidemiologic characteristics of abnormal behaviors (mainly aggressive behavior and non-suicidal self-injury) among Chinese adolescents, and to provide evidence for forming prevention and therapy interventions.

Study design: Multicenter cross-sectional study.

Study sites: Five representative provinces in China, including Heilongjiang (Northern); Anhui (Eastern); Guangdong (Southern); Yunnan (Western) and Hubei (Central).

Sampling and study participants selection: A multistage, random cluster sampling method in each study site was used to select the study participants. First, with the help of local educational bureaus, we selected 9 high schools in each province. Then, in each selected school, we used random digits to choose 2 to 3 classes from each grade (grades 7-9 in junior high schools and graded 10-12 in senior high schools). All students in the selected classes were eligible to participate in the study except for those with severe mental disorders (e.g., severe depression, schizophrenia, paranoid psychosis, and bipolar disorder) who were identified by the head teacher and/or the health care physicians [1, 2]. Consent forms were sent to a total of 15,797 students of 343 classes in 45 public high schools (27 junior high schools and 18 senior high schools) by the head teacher of each selected class to ask for their participation. All the interested students or their guardians (if the student was younger than 14 years) provided written informed consent that was obtained in a manner consistent with the Declaration of Helsinki, before participation in the survey. Of 15,797 students, 78 did not provide the consent form, 21 were absent from school on the day of the survey, and 75 submitted an incomplete questionnaire with at least 15% of the items unanswered. The final sample included 15,623 participants with a response rate of 98.9%.

Data collection: We used a structured questionnaires to collect data during February 18 to October 15, 2015. Trained investigators (teachers and postgraduates) were available at each site to clarify the participants' possible confusion and questions about the structured questionnaire. Participants completed the survey in a single sitting while at school. Before the survey, all participants were informed of the purposes and procedures of the study in detail. All participants were required to complete the anonymous questionnaire independently within 30 to 35 minutes. Completeness of questionnaires was reviewed by investigators before the participants left the site. The questionnaires

were anonymous, and participants did not receive financial compensation.[1, 2]

Measurement of potential confounders

We used a custom designed questionnaire to collect demographic characteristics, familial, and parenting variables.[3] Parenting style was measured by the question of "What kind of parenting style do you think that your main caregivers treat you?.[4] Social support was measured by the 17-item Adolescent Social Support Scale.[5] Loneliness was measured by the revised version of the Loneliness Scale [6]. Psychological resilience was measured through the Resilience Scale for Chinese Adolescents,[7] and emotional management ability was assessed by a 4-items sub-scale of the Emotional Intelligence inventory.[8] All the scales have good internal consistency, with the Cronbach α coefficient in the present study being 0.93, 0.78, 0.76, and 0.78, respectively. The detailed instruction of these scales has been described previously.[1,4]

Data analysis

Frequencies and proportions for categorical variables or mean (SD) for continuous variables were used to describe characteristics of participants and total or subtypes of aggression by study variables. We used the χ^2 test for categorial variables and unpaired *t*-test for continuous variables to compare the distribution between participants of parental migration and non-migration.

Different confounders were adjusted for in 3 binomial logistic regression models. In model 1, we adjusted for the demographic characteristics of participants, including study province, age, sex, and ethnicity. In model 2, we additionally adjusted for confounders of family-level characteristics, including single-parent family, single-child, family income, main caregiver's educational level, parenting styles, and social support. In model 3, we additionally adjusted for psychological confounders, including loneliness, psychological resilience, and emotional management ability. In the sensitivity and subgroup analyses, we adjusted for the most covariates.

We imputed missing data of continuous variables based on mean values and imputed missing data of categorical variables using a separate category as we did in our previous studies.[1,4]

References

1. Tang J, Ma Y, Lewis SP, et al. Association of internet addiction with nonsuicidal self-injury among adolescents in China. *JAMA Netw Open*. 2020;3(6): e206863.

© 2024 Ma Y et al. *JAMA Network Open.*

2. Tang J, Li G, Chen B, et al. Prevalence of and risk factors for non-suicidal self-injury in rural China: Results from a nationwide survey in China. *J Affect Disord*. 2018; 226: 188-195.
3. Ma Y, Li Y, Xie X, et al. The role of depressive symptoms and social support in the association of internet addiction with non-suicidal self-injury among adolescents: a cohort study in China. *BMC Psychiatry*. 2023;23(1):322.
4. Ma Y, Guo H, Guo S, et al. Association of the labor migration of parents with nonsuicidal self-injury and suicidality among their offspring in China. *JAMA Netw Open*. 2021; 4(11): e2133596.
5. Ye Y, Dai X. Development of social support scale for university students. *Chin J Clin Psychol*. 2008; 16(5): 456-458.
6. Li XW, Zou H, Liu Y. Psychometric evaluation of loneliness scale in Chinese middle school students. *Chin Clin Psychology*. 2014; 22(4): 731-733.
7. Hu Y, Gan Y. Development and psychometric validity of the Resilience Scale for Chinese adolescents. *Acta Psychol Sin*. 2008;40(8):902-912.
8. Goleman D. Emotional Intelligence. Bantam Books; 1995.

© 2024 Ma Y et al. *JAMA Network Open.*

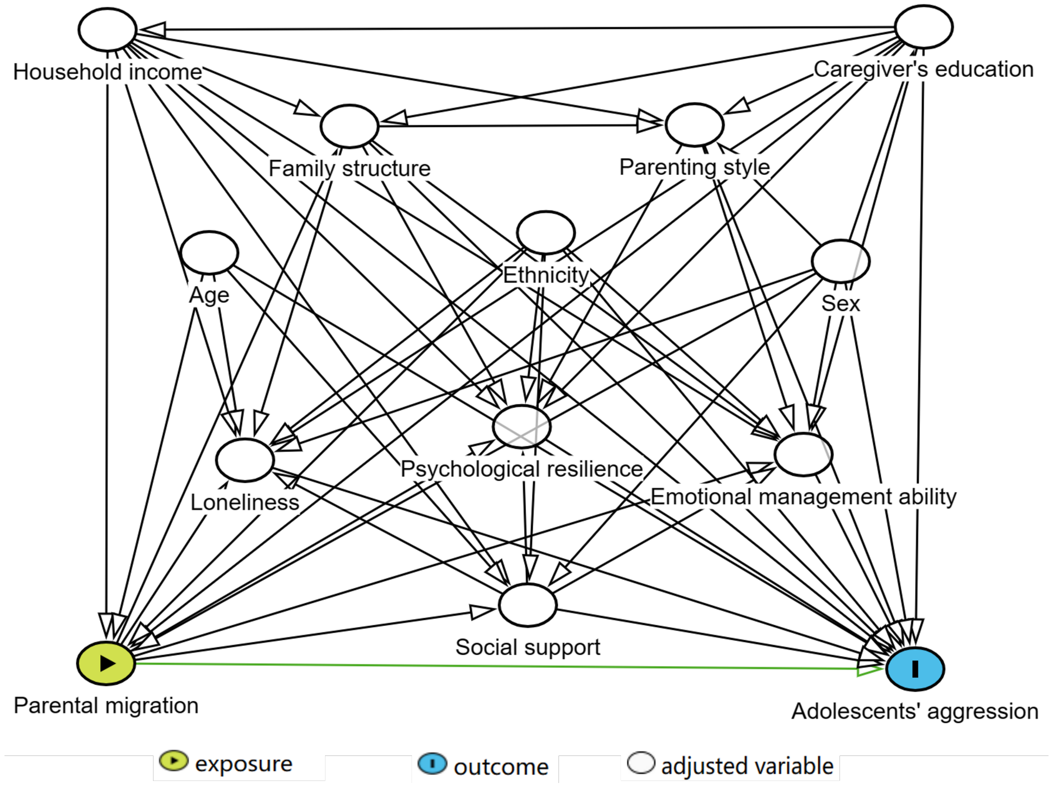

eFigure. Directed Acyclic Graph for the Association Between Parental Migration and Aggression Among Their Offspring

eTable 1. Total and Subtypes of Aggression by Migration Status in Male vs Female Participants

Variable [a]	Male			Female			ROR [d]	*P* value [e]
	N (%)	OR (95%CI) [b]	OR (95%CI) [c]	N (%)	OR (95%CI) [b]	OR (95%CI) [c]		
**Total aggression**								
No migration	799 (16.6)	1 [Reference]	1 [Reference]	640 (14.1)	1 [Reference]	1 [Reference]		
Migration	560 (18.0)	1.10 (1.04-1.19)	1.06 (1.01-1.13)	452 (15.8)	1.15 (1.06-1.25)	1.07 (1.03-1.13)	0.99	0.389
**PA**								
No migration	954 (19.9)	1 [Reference]	1 [Reference]	459 (10.1)	1 [Reference]	1 [Reference]		
Migration	674 (21.7)	1.11 (1.01-1.24)	1.14 (1.03-1.29)	320 (11.2)	1.12 (1.04-1.22)	1.13 (1.05-1.25)	1.01	0.452
**VA**								
No migration	807 (16.8)	1 [Reference]	1 [Reference]	588 (12.9)	1 [Reference]	1 [Reference]		
Migration	505 (16.2)	0.96 (0.85-1.08)	0.92 (0.81-1.05)	383 (13.4)	1.05 (0.91-1-20)	0.96 (0.83-1.11)	0.96	0.334
**IA**								
No migration	900 (18.8)	1 [Reference]	1 [Reference]	857 (18.8)	1 [Reference]	1 [Reference]		
Migration	591 (19.0)	1.02 (0.91-1.14)	0.98 (0.87-1.11)	551 (19.3)	1.03 (0.92-1.16)	0.96 (0.85-1.09)	1.02	0.408
**Anger**								
No migration	637 (13.3)	1 [Reference]	1 [Reference]	777 (17.1)	1 [Reference]	1 [Reference]		
Migration	407 (13.1)	0.98 (0.86-1.12)	0.95 (0.82-1.10)	487 (17.1)	1.00 (0.88-1.13)	0.93 (0.81-1.07)	1.02	0.418
**Hostility**								
No migration	786 (16.4)	1 [Reference]	1 [Reference]	728 (16.0)	1 [Reference]	1 [Reference]		
Migration	539 (17.3)	1.07 (0.95-1.21)	0.99 (0.86-1.13)	510 (17.9)	1.15 (1.01-1.30)	0.97 (0.84-1.12)	1.02	0.420

[a] IA: indirect aggression; PA: physical aggression; VA: verbal aggression. [b] Unadjusted model. [c] Adjusted for provinces, age, ethnicity, single child family, one parent family, educational level of main caregiver, family income, parenting styles and social support, loneliness, psychological resilience, emotional management ability scores.
[d] Calculated by adjusted OR. [e] one-sided *P* value.

© 2024 Ma Y et al. *JAMA Network Open.*

eTable 2. Total and Subtypes of Aggression by Parental Migration Type in Male vs Female Participants

Variable[a]	Male			Female			ROR [d]	*P* value [e]
	N (%)	OR (95%CI) [b]	OR (95%CI) [c]	N (%)	OR (95%CI) [b]	OR (95%CI) [c]		
**Total aggression**								
No migration	799 (16.6)	1 [Reference]	1 [Reference]	640 (14.1)	1 [Reference]	1 [Reference]		
Father migration	350 (19.0)	1.17 (1.06-1.28)	1.12 (1.02-1.23)	248 (14.3)	1.02 (0.87-1.20)	0.98 (0.82-1.17)	1.14	0.096
Mother migration	40 (16.0)	0.95 (0.68-1.35)	1.03 (0.71-1.49)	35 (15.2)	1.10 (0.76-1.59)	1.06 (0.71-1.59)	0.97	0.459
Both parent migration	170 (16.7)	1.01 (0.84-1.21)	0.96 (0.79-1.17)	169 (15.2)	1.23 (1.08-1.38)	1.15 (1.04-1.25)	0.83	0.051
**PA**								
No migration	954 (19.9)	1 [Reference]	1 [Reference]	459 (10.1)	1 [Reference]	1 [Reference]		
Father migration	409 (22.2)	1.15 (1.06-1.26)	1.17 (1.05-1.28)	191 (11.0）	1.10 (0.92-1.32)	1.15 (0.95-1.39)	1.02	0.437
Mother migration	48 (19.2)	0.96 (0.69-1.32)	1.06 (0.75-1.48)	27 (11.7)	1.19 (0.79-1.79)	1.19 (0.77-1.84)	0.89	0.341
Both parent migration	217 (21.4)	1.10 (1.02-1.19)	1.12 (1.04-1.21)	102 (11.4)	1.15 (1.06-1.25)	1.11 (1.03-1.21)	1.01	0.437
**VA**								
No migration	807 (16.9)	1 [Reference]	1 [Reference]	588 (12.9)	1 [Reference]	1 [Reference]		
Father migration	319 (17.3)	1.03 (0.89-1.19)	1.00 (0.86-1.16)	220 (12.7)	0.98 (0.83-1.16)	0.91 (0.76-1.09)	1.10	0.215
Mother migration	40 (16.0)	0.94 (0.67-1.33)	0.95 (0.67-1.36)	28 (12.2)	0.94 (0.62-1.40)	0.87 (0.58-1.33)	1.09	0.376
Both parent migration	146 (14.4)	0.83 (0.69-1.01)	0.85 (0.71-1.03)	135 (15.1)	1.20 (0.98-1.47)	1.07 (0.86-1.33)	0.79	0.058
**IA**								
No migration	900 (18.8)	1 [Reference]	1 [Reference]	857 (18.8)	1 [Reference]	1 [Reference]		
Father migration	362 (19.6)	1.06 (0.92-1.21)	1.03 (0.89-1.18)	314 (18.1)	0.96 (0.83-1.10)	0.92 (0.79-1.07)	1.12	0.143
Mother migration	40 (16.0)	0.82 (0.58-1.17)	0.84 (0.59-1.20)	37 (16.1)	0.83 (0.58-1.19)	0.79 (0.54-1.15)	1.06	0.408
Both parent migration	189 (18.6)	0.99 (0.83-1.18)	0.94 (0.78-1.12)	200 (22.4)	1.25 (1.05-1.48)	1.09 (0.91-1.32)	0.86	0.132
**Anger**								
No migration	636 (13.3)	1 [Reference]	1 [Reference]	777 (17.1)	1 [Reference]	1 [Reference]		

© 2024 Ma Y et al. *JAMA Network Open.*

Father migration	251 (13.6)	1.03 (0.88-1.20)	0.97 (0.82-1.15)	279 (16.1)	0.93 (0.80-1.09)	0.90 (0.76-1.06)	1.08	0.268
Mother migration	32 (12.8)	0.96 (0.66-1.40)	1.02 (0.69-1.53)	34 (14.8)	0.84 (0.58-1.22)	0.82 (0.55-1.22)	1.24	0.224
Both parent migration	124 (12.2)	0.91 (0.74-1.12)	0.90 (0.72-1.12)	174 (19.5)	1.18 (0.98-1.41)	1.02 (0.83-1.25)	0.88	0.208
**Hostility**								
No migration	787 (16.4)	1 [Reference]	1 [Reference]	728 (16.0)	1 [Reference]	1 [Reference]		
Father migration	337 (18.3)	1.14 (0.99-1.31)	1.04 (0.89-1.21)	287 (16.6)	1.05 (0.90-1.22)	0.91 (0.77-1.09)	1.14	0.130
Mother migration	40 (16.0)	0.97 (0.69-1.37)	1.03 (0.71-1.50)	38 (16.5)	1.04 (0.73-1.49)	0.91 (0.61-1.35)	1.13	0.328
Both parent migration	162 (16.0)	0.97 (0.81-1.17)	0.89 (0.73-1.09)	185 (20.7)	1.38 (1.15-1.65)	1.10 (0.89-1.35)	0.81	0.075

[a] IA: indirect aggression; PA: physical aggression; VA: verbal aggression. [b] Unadjusted model. [c] Adjusted for provinces, age, ethnicity, single child family, one parent family, educational level of main caregiver, family income, parenting styles and social support, loneliness, psychological resilience, emotional management ability scores. [d] Calculated by adjusted OR. [e] one-sided *P* value. c, calculated by adjusted OR. d, one-sided *P* value

© 2024 Ma Y et al. *JAMA Network Open.*

eTable 3. Total and Subtypes of Aggression by Parental Migration Stage in Male vs Female Participants

Variable [a]	Male			Female			ROR [d]	*P* value [e]
	N (%)	OR (95%CI) [b]	OR (95%CI) [c]	N (%)	OR (95%CI) [b]	OR (95%CI) [c]		
**Total aggression**								
No migration	753 (16.9)	1 [Reference]	1 [Reference]	603 (14.0)	1 [Reference]	1 [Reference]		
Preschool age (≤ 6y)	260 (17.9)	1.15 (0.98-1.34)	1.03 (0.86-1.22)	235 (16.5)	1.19 (1.01-1.41)	1.00 (0.83-1.22)	1.03	0.412
School age (6y~10y)	231 (16.7)	1.03 (0.87-1.21)	1.04 (0.87-1.24)	169 (14.9)	1.09 (0.91-1.32)	1.09 (0.89-1.35)	0.95	0.368
Adolescence (>10y)	115 (18.9)	1.17 (1.04-1.30)	1.21 (1.06-1.35)	85 (15.6)	1.17 (1.03-1.31)	1.19 (1.06-1.31)	1.02	0.419
**PA**								
No migration	881 (19.8)	1 [Reference]	1 [Reference]	425 (9.9)	1 [Reference]	1 [Reference]		
Preschool age (≤ 6y)	305 (21.0)	1.11 (0.95-1.28)	1.08 (0.92-1.26)	166 (11.6)	1.13 (0.93-1.38)	1.08 (0.87-1.34)	1.00	0.500
School age (6y~10y)	298 (21.5)	1.11 (1.03-1.19)	1.18 (1.02-1.34)	122 (10.7)	1.07 (0.87-1.33)	1.13 (0.90-1.42)	1.04	0.375
Adolescence (>10y)	144 (23.6)	1.16 (1.03-1.30)	1.22 (1.05-1.36)	66 (12.1)	1.21 (1.04-1.37)	1.28 (1.06-1.49)	0.95	0.330
**VA**								
No migration	758 (17.0)	1 [Reference]	1 [Reference]	547 (12.7)	1 [Reference]	1 [Reference]		
Preschool age (≤ 6y)	238 (16.4)	0.98 (0.83-1.15)	0.92 (0.78-1.09)	190 (13.3)	1.00 (0.83-1.20)	0.86 (0.71-1.05)	1.07	0.304
School age (6y~10y)	212 (15.3)	0.91 (0.77-1.08)	0.90 (0.76-1.07)	161 (14.2)	1.12 (0.92-1.36)	1.07 (0.87-1.31)	0.84	0.102
Adolescence (>10y)	104 (17.1)	1.02 (0.80-1.30)	0.98 (0.77-1.26)	73 (13.4)	1.01 (0.76-1.34)	0.99 (0.74-1.33)	0.99	0.479
**IA**								
No migration	847 (19.0)	1 [Reference]	1 [Reference]	811 (18.9)	1 [Reference]	1 [Reference]		
Preschool age (≤ 6y)	282 (19.4)	1.06 (0.91-1.23)	0.98 (0.83-1.15)	289 (20.3)	1.09 (0.93-1.27)	0.97 (0.82-1.15)	1.01	0.466
School age (6y~10y)	250 (18.1)	0.99 (0.84-1.16)	0.99 (0.84-1.17)	198 (17.4)	0.93 (0.78-1.11)	0.89 (0.74-1.07)	1.11	0.200
Adolescence (>10y)	112 (18.4)	0.97 (0.77-1.23)	0.95 (0.75-1.22)	110 (20.2)	1.12 (0.88-1.42)	1.09 (0.85-1.40)	0.87	0.220
**Anger**								
No migration	598 (13.4)	1 [Reference]	1 [Reference]	730 (17.0)	1 [Reference]	1 [Reference]		

© 2024 Ma Y et al. *JAMA Network Open.*

Preschool age (≤ 6y)	193 (13.3)	1.03 (0.86-1.23)	0.93 (0.77-1.13)	243 (17.1)	1.00 (0.85-1.18)	0.87 (0.72-1.04)	1.07	0.311
School age (6y~10y)	173 (12.5)	0.96 (0.79-1.15)	0.96 (0.78-1.16)	193 (17.0)	0.99 (0.83-1.19)	0.98 (0.81-1.19)	0.98	0.442
Adolescence (>10y)	79 (13.0)	0.94 (0.72-1.24)	0.98 (0.73-1.31)	98 (18.0)	1.01 (0.78-1.30)	0.99 (0.75-1.30)	0.99	0.479
**Hostility**								
No migration	732 (16.4)	1 [Reference]	1 [Reference]	677 (15.8)	1 [Reference]	1 [Reference]		
Preschool age (≤ 6y)	269 (18.6)	1.17 (1.00-1.37)	1.01 (0.85-1.20)	267 (18.7)	1.19 (1.01-1.39)	1.05 (0.84-1.20)	0.96	0.3379
School age (6y~10y)	221 (16.0)	0.98 (0.83-1.16)	0.94 (0.78-1.12)	192 (16.9)	1.05 (0.88-1.26)	0.96 (0.78-1.17)	0.98	0.440
Adolescence (>10y)	104 (17.1)	1.04 (0.82-1.33)	1.06 (0.81-1.38)	102 (18.8)	1.24 (0.97-1.59)	1.23 (0.94-1.62)	0.86	0.222

[a] IA: indirect aggression; PA: physical aggression; VA: verbal aggression. [b] Unadjusted model. [c] Adjusted for provinces, age, ethnicity, single child family, one parent family, educational level of main caregiver, family income, parenting styles and social support, loneliness, psychological resilience, emotional management ability scores. [d] Calculated by adjusted OR. [e] one-sided *P* value. c, calculated by adjusted OR. d, one-sided *P* value.

© 2024 Ma Y et al. *JAMA Network Open.*

eTable 4. Odds of Severe Aggression by Parental Migration Status[a]

Variable [b]	Participants, No (%)	Odds ratio (95%CI)	
		Model 1[c]	Model 2 [d]
Total aggression			
No migration	364 (3.9)	1 [Reference]	1 [Reference]
Migration	256 (4.3)	1.08 (1.04-1.12)	1.04 (1.02-1.07)
PA			
No migration	448 (4.8)	1 [Reference]	1 [Reference]
Migration	330 (5.5)	1.16 (1.05-1.26)	1.20 (1.08-1.31)
VA			
No migration	343 (3.7)	1 [Reference]	1 [Reference]
Migration	220 (3.7)	1.01 (0.85-1.20)	0.96 (0.80-1.15)
IA			
No migration	412 (4.4)	1 [Reference]	1 [Reference]
Migration	269 (4.5)	1.03 (0.88-1.20)	1.10 (0.85-1.18)
Anger			
No migration	444 (4.7)	1 [Reference]	1 [Reference]
Migration	278 (4.7)	0.98 (0.84-1.14)	0.93 (0.79-1.10)
Hostility			
No migration	401 (4.3)	1 [Reference]	1 [Reference]
Migration	272 (4.6)	1.09 (0.91-1.25)	0.94 (0.77-1.15)

[a] Severe aggressive behavior was defined as the T-score of total and five subscales of BWAQ≥70T.

[b] IA: indirect aggression; PA: physical aggression; VA: verbal aggression. [c] Unadjusted. [d] Adjusted for provinces, age, ethnicity, gender, single child family, one parent family, educational level of main caregiver, family income, parenting styles and social support, loneliness, psychological resilience, emotional management ability scores.

.

© 2024 Ma Y et al. *JAMA Network Open.*

eTable 5. Odds of Severe Aggression by Parental Migration Type[a]

Variable [b]	Participants, No (%)	Odds ratio (95%CI)	
		Model 1[c]	Model 2[d]
Total aggression			
None	364 (3.9)	1 [Reference]	1 [Reference]
Father	152 (4.3)	1.07 (1.04-1.11)	1.04 (1.02-1.07)
Mother	22 (4.6)	1.05 (0.65-1.70)	1.13 (0.68-1.87)
Both parents	82 (4.3)	1.12 (1.05-1.20)	1.06 (1.02-1.11)
PA			
None	448 (4.8)	1 [Reference]	1 [Reference]
Father	189 (5.3)	1.11 (1.03-1.20)	1.14 (1.05-1.24)
Mother	28 (5.8)	1.23 (0.83-1.82)	1.36 (0.90-2.04)
Both parents	113 (5.9)	1.25 (1.01-1.51)	1.26 (1.03-1.48)
VA			
None	343 (3.7)	1 [Reference]	1 [Reference]
Father	139 (3.9)	1.06 (0.87-1.30)	1.02 (0.83-1.26)
Mother	15 (3.1)	0.85 (0.50-1.43)	0.86 (0.50-1.47)
Both parents	66 (3.5)	0.94 (0.72-1.23)	0.87 (0.66-1.14)
IA			
None	412 (4.4)	1 [Reference]	1 [Reference]
Father	161 (4.5)	1.02 (0.85-1.23)	1.01 (0.83-1.23)
Mother	23 (4.8)	1.09 (0.71-1.68)	1.16 (0.75-1.81)
Both parents	85 (4.5)	1.01 (0.80-1.28)	0.94 (0.73-1.21)
Anger			
None	444 4.8)	1 [Reference]	1 [Reference]
Father	160 (4.5)	0.94 (0.78-1.13)	0.89 (0.73-1.09)
Mother	19 (4.0)	0.83 (0.52-1.32)	0.86 (0.53-1.39)
Both parents	99 (5.2)	1.10 (0.88-1.38)	1.01 (0.79-1.29)
Hostility			
None	401 (4.3)	1 [Reference]	1 [Reference]
Father	162 (4.5)	1.06 (0.85-1.32)	0.92 (0.73-1.17)
Mother	20 (4.2)	1.14 (0.69-1.87)	1.17 (0.90-1.98)
Both parents	90 (4.7)	1.13 (0.86-1.48)	0.92 (0.68-1.23)

[a] Severe aggressive behavior was defined as the T-score of total and five subscales of BWAQ≥70T.

[b] IA: indirect aggression; PA: physical aggressive; VA: verbal aggressive. [c] Unadjusted. [d] Adjusted for provinces, age, ethnicity, gender, single child family, one parent family, educational level of main caregiver, family income, parenting styles and social support, loneliness, psychological resilience, emotional management ability scores.

© 2024 Ma Y et al. *JAMA Network Open.*

eTable 6. Odds of Severe Aggression by Age of Offspring When Parent Initially Migrated[a]

Variable [b]	Participants, No (%)	Odds ratio	
		Model 1 [c]	Model 2 [d]
Total aggression			
No migration	335 (3.8)	1 [Reference]	1 [Reference]
Preschool age (≤6 y)	126 (4.4)	1.15 (0.94-1.21)	1.08 (0.92-1.21)
School age (6-10 y)	104 (4.1)	1.08 (0.87-1.35)	1.08 (0.84-1.39)
Adolescence (>10 y)	55 (4.8)	1.26 (1.06-1.45)	1.14 (1.03-1.25)
PA			
No migration	411 (4.7)	1 [Reference]	1 [Reference]
Preschool age (≤6 y)	150 (5.2)	1.08 (0.89-1.31)	1.05 (0.86-1.30)
School age (6-10 y)	147 (5.8)	1.25 (1.03-1.48)	1.33 (1.08-1.54)
Adolescence (>10 y)	70 (6.1)	1.20 (1.04-1.37)	1.29 (1.09-1.51)
VA			
No migration	318 (3.6)	1 [Reference]	1 [Reference]
Preschool age (≤6 y)	109 (3.8)	1.02 (0.82-1.29)	0.94 (0.75-1.19)
School age (6-10 y)	94 (3.7)	1.04 (0.82-1.32)	1.02 (0.80-1.30)
Adolescence (>10 y)	42 (3.6)	0.87 (0.60-1.26)	0.86 (0.59-1.26)
IA			
No migration	390 (4.5)	1 [Reference]	1 [Reference]
Preschool age (≤6 y)	130 (4.5)	1.02 (0.83-1.26)	0.94 (0.76-1.17)
School age (6-10 y)	107 (4.2)	0.98 (0.79-1.23)	1.00 (0.79-1.25)
Adolescence (>10 y)	54 (4.7)	1.13 (0.83-1.54)	1.17 (0.85-1.60)
Anger			
No migration	418 (4.8)	1 [Reference]	1 [Reference]
Preschool age (≤6 y)	148 (5.1)	1.11 (0.91-1.34)	0.97 (0.78-1.19)
School age (6-10 y)	108 (4.3)	0.91 (0.73-1.14)	0.92 (0.73-1.16)
Adolescence (>10 y)	48 (4.2)	0.82 (0.59-1.15)	0.85 (0.60-1.21)
Hostility			
No migration	370 (4.2)	1 [Reference]	1 [Reference]
Preschool age (≤6 y)	157 (5.5)	1.29 (1.02-1.61)	0.97 (0.76-1.24)
School age (6-10 y)	100 (4.0)	0.92 (0.70-1.20)	0.87 (0.65-1.16)
Adolescence (>10 y)	46 (4.0)	0.96 (0.65-1.41)	1.02 (0.68-1.53)

[a] Severe aggression was defined as the T-score of total and five subscales of BWAQ ≥70T. [b] IA: indirect aggression; PA: physical aggressive; VA: verbal aggressive. [c] Unadjusted. [d] Adjusted for provinces, age, ethnicity, gender, single child family, one parent family, educational level of main caregiver, family income, parenting styles and social support, loneliness, psychological resilience, emotional management ability scores.

© 2024 Ma Y et al. *JAMA Network Open.*

eTable 7. Association of Parental Migration Status With Total and Subtypes of Aggression by Study Location

	OR (95% CI) [a]					
	**PA [b]**	**VA [b]**	**IA [b]**	**Anger**	**Hostility**	**Total aggression**
**Anhui** [eastern]	1.16 (0.95-1.41)	0.99 (0.82-1.21)	1.06 (0.88-1.27)	1.05 (0.85-1.29)	0.91 (0.74-1.12)	1.17 (0.95-1.44)
**Guangdong [**southern]	1.13 (0.86-1.47)	1.11 (0.88-1.38)	0.95 (0.77-1.17)	0.82 (0.65-1.05)	1.10 (0.89-1.36)	1.08 (0.85-1.37)
**Yunnan** [western]	1.28 (1.02-1.60)	1.09 (0.85-1.38)	0.99 (0.79-1.23)	1.26 (1.00-1.59)	1.08 (0.85-1.37)	1.51 (1.20-1.90)
**Heilongjiang** [northern]	1.23 (0.97-1.56)	0.79 (0.62-1.02)	0.89 (0.69-1.13)	0.79 (0.60-1.04)	1.06 (0.81-1.39)	0.98 (0.75-1.27)
**Hubei** [central]	1.07 (0.86-1.32)	0.87 (0.70-1.09)	0.87 (0.72-1.04)	1.03 (0.81-1.30)	1.04 (0.83-1.30)	0.91 (0.72-1.13)

[a] Adjusted for provinces, age, ethnicity, gender, single child family, one parent family, educational level of main caregiver, family income, parenting styles and social support, loneliness, psychological resilience, emotional management ability scores. [b] IA: indirect aggression; PA: physical aggression; VA: verbal aggression.

© 2024 Ma Y et al. *JAMA Network Open.*
