# Supplementary material for: Labor Migration of Parents and Aggression Among Their Offspring in China
Source: JAMA Netw Open. 2024 Feb 8;7(2):e2355315. doi: 10.1001/jamanetworkopen.2023.55315 (PMC10853836; doi:10.1001/jamanetworkopen.2023.55315)
Supplement: Supplement 2. — Data Sharing Statement [file jamanetwopen-e2355315-s002.pdf]

Data Sharing Statement

Ma. Labor Migration of Parents and Aggression Among Their Offspring in China. *JAMA Netw Open*. Published February 08, 2024. doi:10.1001/jamanetworkopen.2023.55315

Data
Data available: Yes
Data types: Deidentified participant data
How to access data: Please contact the corresponding author (Prof Jie Tang: gzy_tangjie@gzhmu.edu.cn) if request for the deidentified participant data
When available: With publication

Supporting Documents
Document types: Statistical/analytic code, Informed consent form
How to access documents: Please contact the corresponding author (Prof Jie Tang: gzy_tangjie@gzhmu.edu.cn) if request for the deidentified participant data
When available: With publication

Additional Information
Who can access the data: investigators whose proposed used of the data has been approved by an independent review committee identified for this purpose.
Types of analyses: For a specified purpose
Mechanisms of data availability: after approval of a proposal, and with a signed data access agreement
